# Quality of life and physical activities of daily living among stroke survivors; cross‐sectional study

**DOI:** 10.1002/nop2.1188

**Published:** 2022-03-09

**Authors:** Sachini Ellepola, Nethuli Nadeesha, Ishara Jayawickrama, Anjali Wijesundara, Nimantha Karunathilaka, Priyamali Jayasekara

**Affiliations:** ^1^ 243144 Department of Nursing & Midwifery Faculty of Allied Health Sciences General Sir John Kotelawala Defence University Colombo Sri Lanka; ^2^ 243144 Department of Clinical Sciences Faculty of Medicine General Sir John Kotelawala Defence University Colombo Sri Lanka

**Keywords:** level of physical activities of daily living, quality of life, stroke

## Abstract

**Aim:**

To examine the relationship between quality of life (QoL), level of physical activities of daily living (PADL) and associated factors among stroke survivors during the acute stage as there is little evidence in Sri Lanka.

**Design:**

We conducted a descriptive cross‐sectional study among conveniently recruited stroke survivors in Sri Lanka (*n* = 134).

**Methods:**

QoL was assessed by the Short Form‐36 (SF‐36), while the PADL was assessed by the Barthel Index (BI). The relationship between SF36 and BI was assessed by Pearson correlation, while Multiple Linear Regression (MLR) was performed to determine the factors associated with QoL and PADL.

**Results:**

The majority of the SF36 domains were below the average level of 50, while BI indicated that most of them belonged to either the severely or totally dependent category. Aphasia, disability, dysarthria, type of stroke, dyslipidaemia, smoking, alcohol consumption, geographical area and income were the associated factors of QoL, while disability of the face and limbs, dysarthria and smoking were the associated factors of PADL (*p* < .05).

## 1 INTRODUCTION

Stroke is defined as “rapidly developing clinical signs of focal (or global) disturbance of cerebral function, lasting more than 24 hr or leading to death, with no apparent cause other than that of vascular origin” (Sacco et al., 2013). The incidence of stroke is comparatively higher in most Asian countries (between 19% and 46%) in comparison to Western countries, and the majority of the incidents are ischaemic compared to haemorrhagic strokes (Wasay et al., 2014). Furthermore, in Sri Lanka, the age‐adjusted prevalence of strokes has been remained constant; 10.6 and 10.4 per 1,000 population in 2009 and 2015 respectively (Gunaratne et al., 2009; Ranawaka & Venketasubramanian, 2021). Moreover, stroke is one of the leading causes of disability among adults in Sri Lanka, and it leads to poor quality of life (QoL) and inability to perform physical activities of daily living (PADL) (Mahesh et al., 2019; Wasay et al., 2014).

Quality of life has been demarcated as an individual's perceptions of their position in life in the context of the culture and value system in which they live and their goals, expectations, standards and concerns (Post, 2014). PADL encompasses basic human actions for caring individuals, including personal care, mobility and eating (Mlinac & Feng, 2016). Furthermore, QoL encompasses individual physical, mental and social well‐being and is also associated with socio‐cultural influence and environmental factors (Waehler et al., 2021). Similarly, people who have an acute stroke are inactive and lonely during hospitalization, and it can lead to several medical comorbidities such as pressure sores, bladder and bowel dysfunction, aspiration pneumonia and deep venous thrombosis (Karklina et al., 2021). Furthermore, the inability to perform PADL due to stroke may affect multisystem dysfunction in the human body (Karklina et al., 2021). Therefore, QoL and PADL are vital determinants for evaluating the status of the disease and improvement of these domains are compatible with the prognosis of stroke and mitigating its complications (Mahesh et al., 2019). Furthermore, many associated factors contribute to strokes, such as hypertension (HTN), diabetes mellitus (DM), dyslipidaemia and smoking. However, developing countries have also contributed to non‐traditional risk factors specific to their region (Wasay et al., 2014). This paper presents the level of QoL, PADL and its associated factors among stroke survivors during their acute stage in Sri Lanka.

## 2 BACKGROUND

Few studies have recently reported a significant association between QoL and PADL (Alotaibi et al., 2021; Haghgoo et al., 2013; Mahesh et al., 2018; Thilarajah et al., 2018; Tsalta‐Mladenov & Andonova, 2021). A cross‐sectional study was conducted among Iranian stroke survivors (*n* = 40) to assess the association between QoL and PADL, and the findings revealed a strong positive correlation between QoL and PADL among people with stroke (*p* < .05) (Haghgoo et al., 2013). Alotaibi et al. (2021) conducted a cross‐sectional study to assess QoL among stroke survivors (*n* = 80) who were followed for 6 months after their diagnosis, and the results revealed that QoL was improved above the average (3.72) of the QoL scale, which ranges from 0 to 5. Furthermore, it showed that age, sex, type of stroke and a recurrent attack was not associated with QoL (Alotaibi et al., 2021). Another prospective study to assess the changes in QoL over 3 months was conducted among stroke survivors in Bulgaria, and its results found that the score of QoL had improved after 3 months, but it was still within the lower reference range (Tsalta‐Mladenov & Andonova, 2021). Furthermore, QoL progressively decreased in age, female sex, lower level of education, high stroke severity scores and anterior circulation stroke (Tsalta‐Mladenov & Andonova, 2021). A prospective cohort study was conducted to assess pre‐ and post‐stroke QoL among stroke survivors in Sri Lanka (Mahesh et al., 2018). The results reported no significant improvement in QoL among stroke survivors after 1 month following hospital discharge. Furthermore, age, female gender, education level, disability and lower health infrastructure were observed as major associated factors in QoL (Mahesh et al., 2018). A systematic review has been conducted to determine factors associated with physical activity followed by a stroke in 2018, and age, gender, physical function, depression, fatigue, self‐efficacy and QoL have been identified as significant factors (Thilarajah et al., 2018).

However, a recent study reported no relationship between QoL and PADL (Kanai et al., 2020). The result showed that QoL was only associated with a number of steps taken by an individual, and it is a valid determinant to estimate the QoL and prognosis of the disease in people with stroke (Kanai et al., 2020). In Sri Lanka, there was a paucity of evidence about QoL, PADL and its associated factors among stroke survivors during their acute stage. However, a similar kind of study assessed only QoL (Mahesh et al., 2018). Furthermore, the associated factors are context‐bound, and there can be timely changes and non‐traditional factors that contribute to the trends in developing countries (Wasay et al., 2014).

Therefore, as an initial step, we conducted a cross‐sectional study to examine the QoL, PADL and its associated factors among stroke survivors during their acute stage (7–10 days followed by the onset of symptoms).

### 2.1 Research question

How does acute stroke influence the QoL and PADL, and which associated factors are involved among stroke survivors in Sri Lanka?

## 3 THE STUDY

### 3.1 Design

A descriptive cross‐sectional design was used for this study.

### 3.2 Method

This study was carried out between January and December 2020 at four major tertiary care hospitals in Sri Lanka. The principal investigators (PIs) instructed ward medical doctors/nurses regarding the inclusion criteria for participants and nurses referred eligible participants to the PIs. Participants diagnosed with acute ischaemic or haemorrhagic stroke (Sacco et al., 2013) and independent before the stroke were selected for the study. All diagnosed transient ischaemic attacks (TIA), patients with stroke who were unconscious or had any diagnosed mental illness, people who had been previously diagnosed with a stroke and people with diagnosed endocrine diseases or inborn errors in metabolism were excluded from the study. We used convenience sampling and met the study's inclusion criteria; there were 134 participants in total.

The instruments used to collect demographic data and the disease‐specific data (DSD) were developed by PIs and modified by two independent Sri Lankan physicians. The DSD was pilot tested with 15 people with stroke and slightly modified again. A clinical examination was performed by two PIs independently and confirmed by the medical record/medical officer at the ward. If there were any discrepancies, clinical findings were confirmed with the in‐charge consultant. All data were collected from participants and secured with an immediate family member. The QoL was assessed through the Short Form 36 (SF36) questionnaire. It is a widely used data collection instrument to calculate health‐related QoL in Sri Lanka and globally (Lins & Carvalho, 2016; Suraweera et al., 2013). The SF 36 consists of eight domains: General health (GH), physical functioning (PF), role limitation‐physical (RLP), Bodily pain (BP), role limitation‐emotional (RLI), vitality (VT), social functioning (SF) and mental health (MH) (Lins & Carvalho, 2016). The raw data in all eight domains were converted to a transformed scale to obtain the actual score of each domain. The maximum scores of PF, RLP, BP, GH, RLI, VT, SF and MH were 20, 04, 10, 20, 20, 08, 03 and 25 respectively (Lins & Carvalho, 2016; Ware et al., 1993).

The PADL was assessed through the Barthel Index (BI), and it has been validated in the Sinhalese language (Lekamwasam et al., 2011). The BI consisted of 10 items, including feeding, bathing, grooming, dressing, bowel and bladder functions, toilet use, transfer (moving between bed and chair), walking on a flat surface and using steps. The total score of BI varies between 0 and 100, where 0 indicates being totally dependent and 100 indicates complete physical independence (Lekamwasam et al., 2011). Furthermore, these values were converted into four categories: totally dependent (0–20), severely dependent (21–60), moderately dependent (61–90) and slightly dependent (91–99) (Lekamwasam et al., 2011). All questionnaires were administered in interview‐administered mode and participants selected a convenient time without hindrance to the ward routines. Additionally, sufficient time is given for answering each question and an adequate break is permitted between each questionnaire.

### 3.3 Analysis

Data were analysed by SPSS 25.0 (SPSS, Chicago, IL, USA). Demographic data and DSD were described using descriptive statistics of frequency/percentage and mean/median along with standard deviation (*SD*)/Inter Quantile Ratio (IQR). The correlation was assessed by Pearson Correlation between the BI (PADL) and each domain of SF36 (QoL), while Multiple Linear Regression (MLR) was used to identify the factors associated with PADL and each domain of QoL without violating MLR's pre‐requisite assumptions. Furthermore, variables that are highly correlated and measure the same construct were not considered when performing MLR. The variance inflation factor (VIF) between variables was assessed and kept the VIF > 5. Durbin–Watson value was taken between 1.5 and 2.5 to identify the first‐order linear auto‐association in linear regression. Categorical variables were converted to dummy variables before performing MLR and the significant level was kept as *p* < .05.

### 3.4 Ethics

Ethics approval was granted by the ethics review committee, Faculty of Medicine, General Sir John Kotelawala Defence University (RP/S/2020/28) and permission were obtained from the respective tertiary care hospitals to conduct the study on their premises. Potential participants were given information letter describing the purposes and processes of the study, their rights and what was expected from them if they have decided to participate. Written informed consent was obtained in their native language. Their privacy, confidentiality and rights were protected throughout the study, and participation was entirely voluntary.

## 4 RESULTS

### 4.1 Demographic characteristics

The mean age was 65.4 ± 11.8 years (*SD*). Most of the participants were males (*n* = 91, 67.9%) and married (*n* = 106, 79.1%). The majority of them (*n* = 122, 91%) were Sinhalese (ethnicity), Buddhist (religion) (*n* = 109, 81.3%) and had their education up to ordinary level (O/L) in school (*n* = 55, 41%). Most of them were retired government employees (*n* = 38, 28.4%), while the average family's monthly income was between 20,001 LKR and 50,000 LKR. Around half of stroke survivors’ primary source of income was from their children and nearly half of them lived with their children (Table 1).

**TABLE 1:** Demographic characteristic of people with acute stroke (*n* = 134)

Characteristic	*n* (%)	Mean (*SD*)
Age		65.4 (11.8) years
Gender		
Male	91 (67.9)	
Female	43 (32.1)	
Marital status		
Married	106 (79.1)	
Single	2 (1.5)	
Widowed	23 (17.2)	
Divorced	3 (2.2)	
Ethnicity		
Sinhala	122 (91.0)	
Tamil	3 (2.2)	
Muslim	9 (6.7)	
Religion		
Buddhist	109 (81.3)	
Hindu	3 (2.2)	
Islam	9 (6.7)	
Catholic	13 (9.7)	
Educational level		
Not at all	2 (1.5)	
Up to grade 5	35 (26.1)	
Up to O/L	55 (41.0)	
Up to A/L	30 (22.4)	
Diploma/degree	12 (9.0)	
Occupation		
Unemployed	32 (23.9)	
Government	38 (28.4)	
Private	26 (19.4)	
Self	38 (28.4)	
Monthly income		
<5,000 LKR	27 (20.1)	
5,001–10,000 LKR	23 (17.2)	
10,001–20,000 LKR	31 (23.1)	
20,001–50,000 LKR	43 (32.1)	
>50,000 LKR	10 (7.4)	
Source of income		
Children	63 (47.0)	
Pension	23 (17.2)	
Spouse	15 (11.2)	
Self‐employment	30 (22.4)	
Other	3 (2.2)	
Living with whom		
Alone	4 (3)	
With spouse	62 (46.3)	
With children	65 (48.5)	
With relative	3 (2.2)	

### 4.2 Disease‐specific data (DSD)—residence type, medical comorbidities, health‐related behaviour and clinical examination

Nearly 80% of the participants lived in a single‐story home with no more than three stairs. Around half of them had a separate bathroom and about 60% had a commode type toilet. The mean distance to the toilet was 12.8 ± 8.7 feet (*SD*). When considering the medical comorbidities of the participant, around 60% of them had been diagnosed with DM and HTN, while only 11.2% and 24.6% were diagnosed with ischaemic heart disease (IHD) and dyslipidaemia. However, just 6% had chronic kidney disease (CKD). Regarding health‐related behaviour, around one‐fourth were smokers with the pack‐years of smoking of 2.9 ± 0.9 and nearly two‐thirds of them were non‐alcoholic.

The mean systolic and diastolic blood pressures were 151.8 ± 22.6 (*SD*) and 86.9 ± 12.9 (*SD*), and around 95% of diagnosed cases were ischaemic strokes while nearly 7% were administered thrombolytic therapy. The middle cerebral artery region (46% of all strokes) was the most affected, followed by the posterior cerebral artery (15%). When considering the disabilities of stroke survivors, most were affected by their left side as 32%, 48%, 48% and 18% respectively in the face, upper limb, lower limb and vision, while almost 44% and 37% had dysarthria and aphasia respectively. The most common type of dysarthria was receptive aphasia (62%), followed by expressive (34%) and global (4%) (Table 2).

**TABLE 2:** DSD characteristics—residence type, medical comorbidities, health‐related behaviour and clinical examination

DSD Characteristics		*n* (%)
Residence condition		
Type of house	Single story	106 (79.1)
	Double story	28 (20.9)
Condition of the bathroom	Separate bathroom	69 (51.5)
	Attached bathroom	65 (48.5)
Type of toilet	Commode	77 (57.5)
	Squatting pan	57 (42.5)
Medical comorbidities		
DM	Yes	78 (58.2)
	No	56 (41.8)
HTN	Yes	83 (61.9)
	No	51 (38.1)
IHD	Yes	15 (11.2)
	No	119 (88.8)
Dyslipidaemia	Yes	33 (24.6)
	No	101 (75.4)
CKD	Yes	8 (6.0)
	No	126 (94.0)
Health‐related behavior		
Smoking	Never smoked	79 (59.0)
	Occasional smoker Active smoker	15 (11.2) 40 (29.9)
Alcohol consumption	None	86 (64.2)
	Occasional	30 (22.4)
	Light	10 (7.5)
	Heavy	8 (6.0)
Clinical examination		
Type of stroke	Ischaemic stroke	127 (94.8)
	Haemorrhagic stroke	7 (5.2)
Thrombolysis (*n* = 127)	Yes	9 (7.0)
	No	118 (93.0)
Infarcted area (*n* = 74)	Middle cerebral	34 (45.9)
	Posterior cerebral	11 (14.9)
	Internal capsule	4 (5.4)
	Other	25 (33.7)
Disability of face	Right	22 (16.4)
	Left	43 (32.1)
	None	43 (32.1)
	None	26 (19.4)
Disability of upper limb	Right	38 (28.4)
	Left	64 (47.8)
	Both	29 (21.6)
	None	3 (2.2)
Disability of lower limb	Right	38 (28.4)
	Left	65 (48.5)
	Both	28 (20.9)
	None	3 (2.2)
Disability of vision	Right	9 (6.7)
	Left	24 (17.9)
	Both	5 (3.7)
	None	96 (71.6)
Dysarthria	Present	59 (44.0)
	None	75 (56.0)
Aphasia	Present	49 (36.6)
	None	85 (63.4)

### 4.3 Relationship in QoL and PADL

The mean scores of the SF 36 domains are shown in Table 3. Mean scores of PF, RLP, GH and RLE were below the average score of 50 and the maximum score of SF‐36 was 63.3 ± 19.1, which belongs to the BP domain (Table 3). The mean score of BI was 42.9 ± 14.9 (*SD*), and the mean score was below the average score of 50, and nearly two‐thirds of them belonged to the severely or totally dependent category (Table 4). Furthermore, there was a significant positive correlation between all domains of SF 36 and BI (*p* < .05) (Table 5).

**TABLE 3:** Scores of SF36 domains

SF 36 domains	*n* = 134	
	Mean	*SD*
PF	21.9	5.5
RLP	23.6	10.1
BP	63.3	19.1
GH	44.7	15.9
VT	51.6	13.6
SF	59.2	15.6
RLE	32.5	14.3
MH	60.0	18.3

**TABLE 4:** Categories of BI

Categories	(*n* = 134)	
	*n* (%)	Range of scores
Totally dependent	57 (42.5)	0–20
Severely dependent	29 (21.6)	21–60
Moderately dependent	36 (26.9)	61–90
Slightly dependent	12 (9)	91–99

**TABLE 5:** Correlation between SF36 domains and BI (*n* = 134)

Parameters	Correlation coefficient (*r*)	*p* value
BI × PF	.843	.001
BI × RLP	.453	.001
BI × BP	.388	.001
BI × GH	.710	.001
BI × VT	.536	.001
BI × SF	.425	.001
BI × RLE	.430	.001
BI × MH	.436	.001

Note

Pearson correlation.

### 4.4 Factors associated with QoL and PADL

The factors of aphasia, disability of face, disability of vision, disability of lower limb, dysarthria, type of stroke, type of caregiver, dyslipidaemia, smoking, alcohol consumption, geographical area and monthly income were the significant determinants contributed as associated factors of QoL (SF 36), while disability of face, disability of lower limb, dysarthria and smoking, were the significant determinants of PADL (BI) (*p* < .05). Furthermore, in QoL, around 28.4%, 8.7%, 22.0%, 21.4%, 22.7%, 24.8%, 37.5% and 65.4% of the variance can be explained by observed variables in the domains of PF, RLP, BP, GH, VT, SF, RLE and MH respectively. Moreover, for BI, about 34.1% of the variance can be explained by the observed variables in this study (Table 6).

**TABLE 6:** Factors associated with QoL and PADL

Determinant	Standardized beta coefficient	*p*	Adjusted *R* ^2^	*F*
QoL (PF)				
Constant		.001	.284	8.8
Aphasia	0.27	.003		
Disability of lower limb	−0.24	.005		
Dysarthria	0.25	.005		
Dyslipidaemia	−0.18	.039		
QoL (RLP)				
Constant		.425	.087	10.0
Aphasia	0.31	.002		
QoL (BP)				
Constant		.001	.220	4.9
Aphasia	0.36	.001		
Disability of vision	0.27	.003		
QoL (GH)				
Constant		.001	.214	9.9
Disability of face	−0.33	.000		
Smoking	−0.18	.042		
QoL (VT)				
Constant		.001	.227	5.5
Aphasia	0.32	.001		
Disability of face	−0.28	.003		
QoL (SF)				
Constant		.003	.248	9.1
Aphasia	0.29	.002		
Type of stroke	0.26	.003		
Alcohol consumption	−0.24	.007		
Disability of face	−0.21	.024		
QoL (RLE)				
Constant		.001	.375	9.4
Disability of face	−0.52	.002		
Geographical area	−0.34	.032		
QoL (MH)				
Constant		.001	.654	8.6
Aphasia	0.50	.001		
Monthly income	0.40	.002		
Disability of face	−0.25	.048		
PADL (BI)				
Constant		.001	.341	8.0
Disability of face	−0.33	.004		
Smoking	−0.23	.045		
Disability of lower limb	−0.29	.011		
Dysarthria	−0.27	.017		

## 5 DISCUSSION

The findings of this study indicate that both QoL and PADL in people with acute stroke are severely or totally dependent and below the average level respectively. Furthermore, there was a positive correlation between all QoL domains and the PADL score, and a few domains of QoL, such as PF and GH, had a strong relationship with PADL (*p* < .05).

Stroke is a disease that affects the central nervous system. The primary purpose of stroke treatment is to improve a patient's level of disability to facilitate functional independence and QoL (Winstein et al., 2016). Most related studies further confirmed that PF and GH were the most affected domains of QoL among stroke survivors, and slight improvement of PF and GH subdomains may directly influence QoL irrespective of the stroke stage, either acute or chronic (Haghgoo et al., 2013; Kim et al., 2014; Mahesh et al., 2018; Whitiana et al., 2017). Generally, PADL is the activities of eating, passing urine, bathing and dressing that facilitate accomplishing daily routines (Kanai et al., 2020). However, most stroke survivors were totally or severely dependent on PADL in implementing daily practices (Haghgoo et al., 2013; Whitiana et al., 2017). Therefore, as confirmed by the present findings, QoL and PADL were inter‐connected and were observed direct association among stroke survivors. However, the deficiency of QoL is not permanent and gradually recover over time. QoL could not much improve 1 month after the stroke, and this period is mainly impaired both QoL and PADL and needed support from family and community (Mahesh et al., 2018). Even after 3 months, QoL stands below the average, but significant improvement can be observed (Tsalta‐Mladenov & Andonova, 2021). After 6 months, QoL reaches above average with proper medical management and social and psychological support (Alotaibi et al., 2021; Kielbergerova et al., 2015). These predictions and timelines should be included in the patient's education programme and encourage everyone to set goals, keep hopes and enhance self‐confidence among the vulnerable and their relations.

The present study has observed some DSD clinical features as associated factors for QoL that are presence or absence of aphasia, type of stroke, disability of the face, upper and lower limbs and dysarthria among acute stage of stroke survivors. Similarly, Mahesh et al. (2018) also reported that disability of face and limb were associated factors in QoL among stroke survivors. Furthermore, well‐known traditional factors such as smoking, HTN, IHD, dyslipidaemia and DM are associated with QoL and PADL in developing countries and are compatible with the present findings (Wasay et al., 2014). The present study has identified a few non‐traditional associated factors, such as geographical regions, alcohol consumption and monthly income. Furthermore, lower infrastructure conditions in the health care sector and the education level of survivors are also significant predictors of QoL (Mahesh et al., 2018). However, some non‐traditional factors did not align with present findings, particularly in geographical areas for QoL among stroke survivors in Australia. It revealed that recommended patient care was comparatively poor in rural regions compared to urban areas (Dwyer et al., 2021). Therefore, expanding the health care delivery system, continuous educational programmes on HTN, DM, dyslipidaemia and healthy behaviour may mitigate recurrent stroke attacks and enhance the QoL.

Our study revealed that disability of limbs, dysarthria and smoking were associated with PADL among stroke survivors during their acute stage. Additionally, Pei et al. (2016) showed that stroke frequency, type of stroke, nutritional status, age and financial status were also predominant predictors for PADL. Furthermore, a recent systematic review reported that age, gender, physical function, depression, fatigue and self‐efficacy were considered associated factors for physical activity among stroke survivors (Thilarajah et al., 2018). However, age and sex were not the significant predictors for PADL in the present study, and a few variables, such as depression, fatigue and self‐efficacy, were not assessed in the present study. Therefore, performing and monitoring PADL is closely related to a quick return to social life and nursing professionals play a vital role. Thus, functional rehabilitation interventions may be essential to solve limited mobility and overcome physical, psychological and social problems (Kim et al., 2014). These interventions may enhance the vulnerable's functional autonomy and reduce the risk of new cerebrovascular events leading to stroke (Belfiore et al., 2018).

### 5.1 Limitations

This research has a few limitations. In a case‐controlled study, the findings of the results compared with people who are not currently diagnosed with stroke but are at high risk of stroke would help us interpret the more valid and reliable results. There was a bias towards selecting the participants by convenient sampling and generalizing the findings to other settings. Furthermore, a prospective longitudinal cohort study is warranted to see how QoL and PADL change over time.

## 6 CONCLUSION

- The findings outlined that most strokes were ischaemic, and both QoL and PADL were affected during the acute stage.
- Most stroke survivors were below the average of QoL and belonged to the totally or severally dependent category in the PADL. Furthermore, a higher level of QoL in each domain indicates a higher level of PADL. However, the physical health and general health domains of QoL were strongly affected by PADL in comparison to other domains of QoL.
- Moreover, factors such as monthly income, geographical area and education level were identified along with traditionally associated factors of QoL and PADL. In addition, the presence of aphasia, dysarthria and disability of the face, upper and lower limbs were observed as associated factors in the acute stage of stroke.
- These results indicate that improvement in PADL accelerates QoL and their confidence to go back to everyday life, even during the acute stage of the disease. Further, this finding can be incorporated into medical and nursing clinical practice to estimate the level of prognosis of the individual followed by an acute stroke.

## AUTHOR CONTRIBUTIONS

Study design—Nimantha Karunathilaka, Sachini Ellepola, Nethuli Nadeesha, Ishara Jayawickrama, Priyamali Jayasekara. Data collection—Nimantha Karunathilaka, Sachini Ellepola, Nethuli Nadeesha, Ishara Jayawickrama, Priyamali Jayasekara, Wijesundara. Data analysis—Nimantha Karunathilaka, Sachini Ellepola, Nethuli Nadeesha, Ishara Jayawickrama, Priyamali Jayasekara. Manuscript writing—Nimantha Karunathilaka, Sachini Ellepola, Nethuli Nadeesha, Ishara Jayawickrama, Priyamali Jayasekara, Wijesundara

## Data Availability

The data that support the findings of this study are available on request from the corresponding author. The data are not publicly available due to privacy or ethical restrictions.
